# Comparative Analysis of Acinetobacters: Three Genomes for Three Lifestyles

**DOI:** 10.1371/journal.pone.0001805

**Published:** 2008-03-19

**Authors:** David Vallenet, Patrice Nordmann, Valérie Barbe, Laurent Poirel, Sophie Mangenot, Elodie Bataille, Carole Dossat, Shahinaz Gas, Annett Kreimeyer, Patricia Lenoble, Sophie Oztas, Julie Poulain, Béatrice Segurens, Catherine Robert, Chantal Abergel, Jean-Michel Claverie, Didier Raoult, Claudine Médigue, Jean Weissenbach, Stéphane Cruveiller

**Affiliations:** 1 Génomique Métabolique, CNRS UMR8030, CEA–Institut de Génomique-Genoscope, Evry, France; 2 Service de Bactériologie-Virologie, Hôpital de Bicêtre, Assistance Publique/Hôpitaux de Paris, Faculté de Médecine Paris-Sud, Université Paris XI, Kremlin-Bicêtre, France; 3 Unité des Rickettsies, CNRS UMR6020, Faculté de Médecine, Université de la Méditerranée, Marseille, France; 4 Information Génomique et Structurale, CNRS UPR2589, IBSM, Marseille, France; University of Liverpool, United Kingdom

## Abstract

*Acinetobacter baumannii* is the source of numerous nosocomial infections in humans and therefore deserves close attention as multidrug or even pandrug resistant strains are increasingly being identified worldwide. Here we report the comparison of two newly sequenced genomes of *A. baumannii*. The human isolate *A*. *baumannii* AYE is multidrug resistant whereas strain SDF, which was isolated from body lice, is antibiotic susceptible. As reference for comparison in this analysis, the genome of the soil-living bacterium *A. baylyi* strain ADP1 was used. The most interesting dissimilarities we observed were that i) whereas strain AYE and *A. baylyi* genomes harbored very few Insertion Sequence elements which could promote expression of downstream genes, strain SDF sequence contains several hundred of them that have played a crucial role in its genome reduction (gene disruptions and simple DNA loss); ii) strain SDF has low catabolic capacities compared to strain AYE. Interestingly, the latter has even higher catabolic capacities than *A. baylyi* which has already been reported as a very nutritionally versatile organism. This metabolic performance could explain the persistence of *A. baumannii* nosocomial strains in environments where nutrients are scarce; *iii*) several processes known to play a key role during host infection (biofilm formation, iron uptake, quorum sensing, virulence factors) were either different or absent, the best example of which is iron uptake. Indeed, strain AYE and *A. baylyi* use siderophore-based systems to scavenge iron from the environment whereas strain SDF uses an alternate system similar to the Haem Acquisition System (HAS). Taken together, all these observations suggest that the genome contents of the 3 Acinetobacters compared are partly shaped by life in distinct ecological niches: human (and more largely hospital environment), louse, soil.

## Introduction

Although low-grade pathogens in humans, human-adapted *Acinetobacter* species mainly belong to the *A. baumannii-A. calcoaceticus complex* are of growing interest due to increased incidence of multidrug resistance (MDR) [1]. *A. baumannii* strains are isolated in up to 1% of nosocomial infections mostly from immunocompromised patients hospitalized in intensive care units. Although *A. baumannii* isolates are commonly found in clinical environment, Acinetobacters are mostly free-living saprophytes ubiquitously found in nature (soil, water, sewage). *Acinetobacter* species have been also identified in small-size living organisms (body lice, fleas and ticks) that are potential vectors for infection transmission [2].

The genus *Acinetobacter* consists of strictly aerobic, Gram-negative coccobacillary rods, non-motile, catalase positive, oxidase negative and growing at 20°–30°C on usual laboratory culture media. g-proteobacteria classified as members of the genus *Acinetobacter* have a long history of taxonomic changes moving from the family *Neisseriaceae* to the family *Moraxellaceae*. Various methods (DNA-DNA hybridization [3], biotyping [4], Amplified Ribosomal DNA Restriction Analysis [5], AFLP [6]) now make it possible to distinguish 32 different species within the genus *Acinetobacter*, 17 of which have officially been given names. Nevertheless, unambiguous species identification may be difficult in particular for some genetically closely related species. Standard tests indeed fail to distinguish *A. baumannii*, *A. calcoaceticus* and the unnamed genomic species 3 and 13TU (the *A. calcoaceticus-A. baumannii* complex [7]).

Natural competence as well as high metabolic capacities have been reported in several *Acinetobacter* species making those species very attractive for environmental and biotechnological use [8]. For example, *Acinetobacter baylyi* ADP1 is highly competent and may grow on a large variety of compounds [9].

Two *Acinetobacter baumannii* strains (AYE and SDF) were initially sequenced in a clinical/medical context. Since the primary goal of the project was to identify the complete repertoire of genes involved in resistance to various antibiotics [10], much of our effort was concentrated on a resistance island of *ca.* 86-kb long which has been uncovered in the genome of the multi-drug resistant human clinical isolate strain AYE responsible for a nationwide outbreak in France in 2001. In contrast, this resistance island was not present in *A. baumannii* strain SDF associated with human body louse, partly explaining its susceptibility to antibiotics.

A few years ago, we published the complete genome of another Acinetobacter (*A. baylyi* ADP1 [9]) for which human experts have performed the annotation process. This complete annotation set therefore appeared to be a strong basis for fast and accurate annotation of the two *A. baumannii* strains.

The availability of 3 complete genome sequences from members of the same genus (*i.e.* AYE, SDF and *A. baylyi* ADP1 hereafter referred as *A. baylyi*) prompted us to compare them in a more general context. Here we provide a detailed comparison of the three genomes that highlights differences that might reflect adaptation to a specific environment. The in-depth analysis of gene contents reveals that though the three organisms share a large fraction of genes, large differences exist. For instance, we show that strain AYE has high catabolic and drug/metal resistance capacities whereas strain SDF is riddled with numerous prophage regions and Insertion Sequences (ISs). Moreover, the example of iron uptake illustrates the diverse strategies used by *Acinetobacter* to thrive in various environmental conditions. These features emphasize the diversity of the *Acinetobacter* genus. We conclude this report with an analysis of potential virulence factors.

## Results and Discussion

### Three *Acinetobacter* genomes: general features and taxonomic considerations

The genome of the *A. baumannii* isolates is made of a single chromosome and several plasmids (4 and 3 for AYE and SDF, respectively). In *A. baylyi*, no plasmid was detected [9]. Strain AYE has the largest chromosome (ca. 3.9 Mb versus 3.4 Mb for strain SDF and 3.6 Mb for *A. baylyi*; Table 1). The GC-content of the three sequences is around 40%, a value corresponding to that reported for other members of the *Acinetobacter* genus.

**Table 1:** Comparison of the general features of the 3 *Acinetobacter* chromosomes.

	*Acinetobacter baylyi* ADP1	*Acinetobacter baumannii* AYE	*Acinetobacter baumannii* SDF
Size (Mb)	3,6	3,9	3,4
Plasmids	none	4	3
GC-content (%)	40,4	39,4	39,2
CDSs [Pseudogenes]	3279 [12]	3590 [37]	2903 [272]
ISs [Pseudogenes]	13 [ 8]	33 [0]	428 [145]
Names	6 [1] IS1236	21 ISAba1, 3 IS10A, 2 IS26, 1 IS15DI	217 [76] ISAba6, 206 [68] ISAba7
Average gene size (bp)	962	951	930
Protein Coding Density (%)	87,34	86,13	78,70
Protein with predicted function (%)	63,76	62,53	64,94
Conserved hypothetical proteins (%)	27,32	30,03	28,03
Hypothetical proteins (%)	8,92	7,44	7,03
tRNA	76	72	64
rRNA clusters	7	6	5
Phage regions	2	6	8
Repeated regions (%)	3,64	5,14	17,21

Although tRNAs for each of the 20 amino acids were found in both *A. baumannii* strains AYE and SDF, the latter has a lower copy number of tRNAs for eight amino acids namely Ala, Arg, Asp, Gln, Ile, Ser, Tyr and Val. The largest differences were found for tRNA-Asp (a single copy instead of three) and for tRNA-Gln (2 copies instead of 4 in AYE and 5 in *A. baylyi*). Dolzani and colleagues [11] showed that the number of rRNA operons may vary between 5 and 7 for the *Acinetobacter* species. Confirming these data, strain AYE possessed 6 rRNA clusters whereas strain SDF contained only 5. Like in the case of tRNAs, the missing rRNA cluster may have been lost during one of the numerous recombination events probably mediated by Insertion Sequences (ISs; see below). As reported in the literature, the 16S–23S Intergenic Spacer Region (16S–23S ISR) of *A. baylyi* strain ADP1 rRNA clusters is of variable length and probably resulted from recombination between rRNA operons [12]. This does not seem to be a feature shared by species belonging to the *Acinetobacter* genus since rRNA clusters 16S–23S ISRs in both *A. baumannii* strains are of constant length (663 bp). Strain SDF's species status was uncertain because this strain has no b-lactamase activity and is unable to grow at 44°C, two features characterizing the *A. baumannii*-*A.calcoaceticus* complex. However, phylogenetic reconstruction based on *Acinetobacter spp.* 16S–23S ISRs currently available in Genbank, clearly placed strain SDF as sister group of other *A*. *baumannii* strains (Figure S1). Furthermore, we found in strain SDF a tiny fragment (24 amino acids) corresponding to the C-terminus of *ampC* gene that shared the same chromosomal context as strain AYE *ampC*. In addition, this fragment is located next to one IS*Aba6* copy implying that *ampC* gene has been disrupted during evolution of this strain. This latter finding is further evidence that SDF is a member of *A. baumannii* species.

### Diversity of *Acinetobacter species*: core genome vs. flexible gene pool

**Figure 1:**
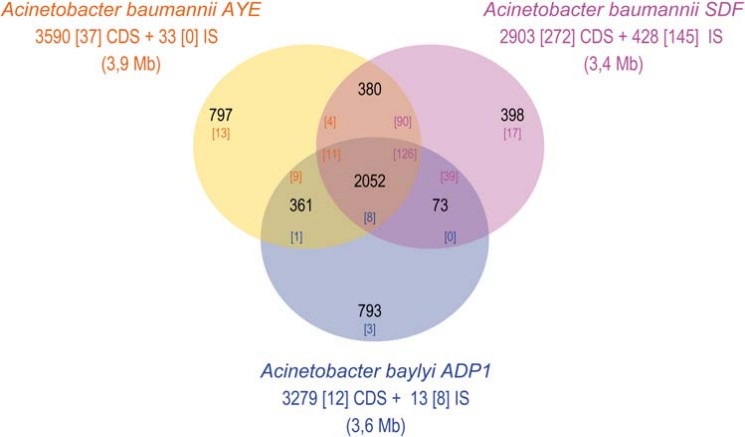
Comparison of gene content of *A. baylyi* ADP1, *A. baumannii* AYE and *A. baumannii* SDF chromosomes. Putative orthologs are defined as genes showing a minimum of 40% identity and a ratio of 0.8 of the length of the smallest protein, or as two genes included in a synteny group. The intersections between the three circles give the number of genes found in the 2 or 3 compared species. Genes outside these areas are specific to the corresponding organism. The total number of annotated genes is also given under each species name. Figures in brackets indicate the number of pseudogenes.

Comparative analyses of the gene content of the three strains define two distinct gene pools: a core genome, the set of orthologous genes shared by the three strains and a flexible gene pool, the sets of genes that either are common to two species out of three or that do not have any significant homolog in any of the two other species (Figure 1). The candidate core genome contains 2,052 genes corresponding to 57.2%, 70.7% and 62.6% of the DNA sequences of *A*. *baumannii* strain AYE, strain SDF and *A. baylyi*, respectively. The sizes of the flexible gene pools may be correlated with different life styles in distinct ecological niches. Even though 361 genes are shared only by AYE and *A. baylyi*, each species possesses almost 800 specific genes. These figures may reveal a high capacity of adaptation in fluctuating environmental conditions. Consistent with life in a less variable environment (hematophage gut), the amount of specific genes in SDF is significantly lower (n = 398; see Figure 1).

**Figure 2:**
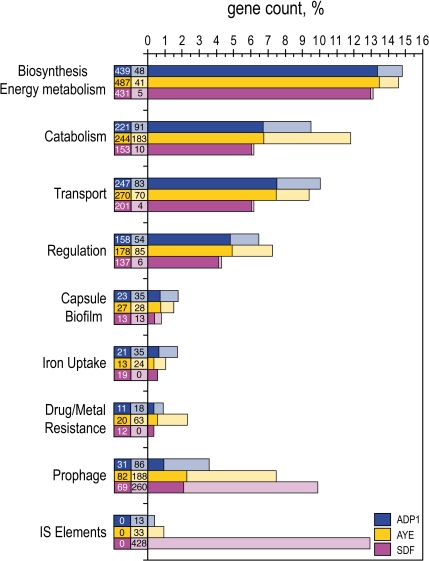
Gene content comparison of the three *Acinetobacter* chromosomes according to nine functional categories. The proportions of specific genes which have no ortholog in the two other strains are indicated by light colors. Genes having an ortholog in at least one of the other 2 species were regarded as non specific (dark colors). Absolute gene count for each subclass is reported in boxes at bars bottom. Two genes were considered as orthologs when their respective product shares more than 40% identity over more than 80% of their length.

For the three species under study, genes were assigned to various functional categories (Figure 2). At first sight, some of the categories display peculiar patterns. Considering *A. baumannii* AYE, 42% of the genes dedicated to catabolic functions and two third of genes involved in drug/metal resistance are specific. In turn in strain SDF, 80% of prophagic genes and all ISs are specific to this organism. The specificity of strain SDF mainly resides in its extrachromosomal elements content whereas strain AYE has pronounced capacities in catabolism and drug/metal resistance which may explain its successful adaptation to the hospital environment (see below).

**Figure 3:**
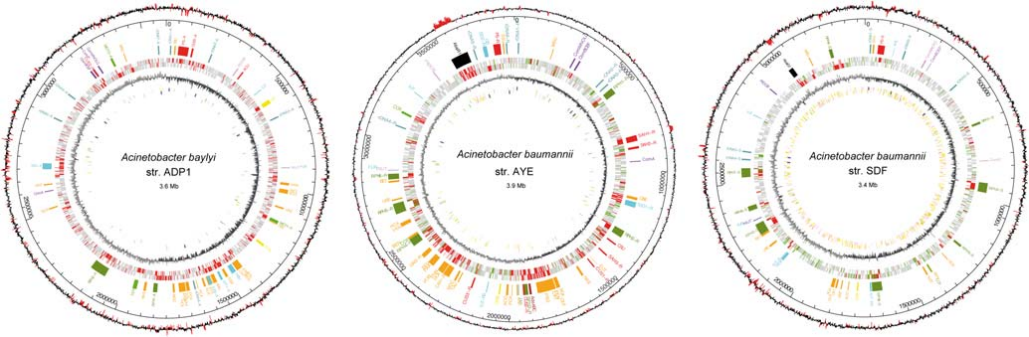
Circular representation of the three *Acinetobacter* genomes (ADP1, left; AYE, middle; SDF, right). Circles display from the outside in: (1) GC deviation (mean GC content in a 1kb window–overall mean GC). Red areas indicate that deviation is higher than 1.5 SD. (2) Location of various genomic regions. (3) Gene specificity (grey, *Acinetobacter spp.* core genome; green, *A. baumannii* core genome; red, strain specific genes). (4) GC skew (G+C/G−C using a 1kb sliding window). (5) Pseudogenes (yellow), ISs (pink), tRNA (green) and rDNA (blue).

IS elements in the three strains were strikingly different (Table 1). Strain SDF has by far the largest number of IS elements made of two novel elements (IS*Aba6* and IS*Aba7*) in about equal proportions and evenly distributed along the chromosome (Figure 3). Those elements belong to IS*982* and IS*5* families, respectively. It is very likely that these IS elements have significantly contributed to the genome size reduction, especially via recombination events and gene disruptions. Twenty-one copies of IS*Aba1* (IS*4* family) were detected in the genome of the human strain *A. baumannii* AYE. The IS element IS*Aba1* has been recently identified from human isolates of *A. baumannii* [13]. Although its role is unclear, it has been demonstrated to contribute at least to overexpression of the naturally-occurring ß-lactamase *ampC* gene leading to high-level resistance to ß-lactams and to overexpression of acquired ß-lactamases genes [13]. Whereas no *ISAba1* copy was identified on any of the plasmids of isolate AYE, chromosomal copies showed no site integration preference (Figure 3). It is important to note that although IS*Aba1* is supposed to be “customized” for *A. baumannii* [14], this element is not present in strain SDF genome.

DNA acquisition/exchanges in bacteria can occur between organisms of the same or different species via one of three mechanisms: conjugative transfer, phage transduction, or natural transformation. The last mechanism relies on the ability of cells to take up and stably maintain large pieces of “alien” DNA. This phenomenon called “natural competence” has been extensively studied in two Gram-negative soil living bacteria, namely *A. baylyi* and *Pseudomonas stutzeri* (see for instance [15]). Besides their involvement in biofilm formation (see below), some components related to type IV pili biogenesis play an important role in natural transformation process [16]. This is consistent with the fact that free extracellular DNA itself may trigger biofilm formation [17]. Almost all genes previously identified in the two models (*com* and *pil* genes) have been found in *A. baumannii* strain AYE whereas their counterparts are either absent or disrupted in strain SDF, suggesting that the latter strain is not competent for natural transformation (Table 2). Several putative prophage regions were roughly delimited in both *A. baumannii* genomes (six for AYE and eight for SDF; Table 2). Though not exceptional, these regions constitute a sizable part of both genomes accounting for 5.1% and 6.7% of whole chromosomes of strain AYE and SDF, respectively. Notably, all prophage regions detected were not similar (within and between genomes; Table 2) as suggested by very low similarities between well-known phage markers and by the obvious absence of synteny between the three genomes. Even though plasmids can be gained via natural transformation, a more specific process of plasmid acquisition is conjugative transfer. In contrast to *A. baylyi*, strains AYE and SDF possess several plasmids (4 and 3 respectively) on which no gene involved in resistance and/or virulence has been characterized. Nevertheless, putative mobilization systems MobS/MobL for DNA exchange and putative toxin/antitoxin systems for plasmid maintenance are present.

**Table 2:** Genomic regions found in the 3 *Acinetobacter* species.

Label	*Acinetobacter baylyi* ADP1	*Acinetobacter baumannii* AYE	*Acinetobacter baumannii* SDF
ACU	ACIAD0387-ACIAD0390, 4.95 kb	-	-
CSU	-	ABAYE1319-ABAYE1324, 6.03 kb	-
CUS1-A	ACIAD0119-ACIAD0124, 7.26 kb	-	-
CUS1-R	-	ABAYE1470-ABAYE1473, 4.79 kb	-
CUS2-A	ACIAD3332-ACIAD3337, 7.03 kb	-	-
CUS2-R	-	ABAYE1856-ABAYE1860, 6.07 kb	-
CUS3′-R	-	ABAYE2132-ABAYE2138, 6.24 kb, pseudo	-
PS-A	ACIAD0057-ACIAD0105, 47.43 kb	-	-
PS-R	-	ABAYE3795-ABAYE3824, 34.31 kb	-
PS-S	-	-	ABSDF0059-ABSDF0083, 29.28 kb
SAH1-R	-	ABAYE0792-ABAYE0794, 28.13 kb	-
SAH2-R	-	ABAYE0821-ABAYE0821, 10.11 kb	-
SAH3-R	-	ABAYE1394-ABAYE1397, 5.98 kb	-
COB	-	ABAYE1990-ABAYE1995, 5.35 kb	ABSDF1877-ABSDF1884, 4.71 kb, pseudo
CYA	ACIAD1276-ACIAD1282, 9.08 kb	-	-
LIP	ACIAD0566-ACIAD0586, 17.72 kb	-	-
ACO	ACIAD1014-ACIAD1022, 9.62 kb	ABAYE1943-ABAYE1950, 9.49 kb	ABSDF1932-ABSDF1934, 2.48 kb, pseudo
ALN	ACIAD1614-ACIAD1622, 76.66 kb	-	-
MSU	ACIAD3470-ACIAD3472, 2.37 kb	ABAYE0181-ABAYE0183, 2.40 kb	ABSDF3432-ABSDF3435, 3.27 kb
SSU	ACIAD0034-ACIAD0038, 4.88 kb	ABAYE3840-ABAYE3844, 4.83 kb	ABSDF0033-ABSDF0038, 5.84 kb
ANT	ACIAD2669-ACIAD2672, 4.58 kb	ABAYE1896-ABAYE1900, 4.46 kb	ABSDF1968-ABSDF1971, 3.85 kb, pseudo
ARE	ACIAD1428-ACIAD1431, 5.71 kb	-	-
BEN	ACIAD1433-ACIAD1440, 8.96 kb	ABAYE2553-ABAYE2561, 9.40 kb	ABSDF1496-ABSDF1498, 2.90 kb, pseudo
BET	ACIAD1008-ACIAD1012, 7.90 kb	ABAYE2864-ABAYE2868, 7.92 kb	ABSDF2436-ABSDF2440, 7.91 kb
CAT	ACIAD1442-ACIAD1451, 9.76 kb	ABAYE1714-ABAYE1721, 7.50 kb	ABSDF1993-ABSDF2000, 7.02 kb
HCA	ACIAD1720-ACIAD1728, 11.06 kb	ABAYE2626-ABAYE2635, 11.66 kb	ABSDF1411-ABSDF1412, 2.02 kb, pseudo
CBE	-	ABAYE1061-ABAYE1069, 12.07 kb	-
DSZ	ACIAD1503-ACIAD1512, 11.11 kb	-	-
DCA	ACIAD1684-ACIAD1697, 14.71 kb	ABAYE2299-ABAYE2315, 17.94 kb	-
EUT	-	ABAYE1457-ABAYE1456, 5.40 kb	-
MDC	ACIAD1753-ACIAD1762, 8.26 kb	ABAYE2224-ABAYE2233, 8.31 kb	-
NAS	ACIAD1908-ACIAD1914, 10.47 kb	ABAYE1541-ABAYE1546, 9.32 kb	ABSDF1522-ABSDF1524, 5.89 kb, pseudo
NAL	ACIAD1565-ACIAD1581, 16.81 kb	-	-
POB	ACIAD1717-ACIAD1719, 3.51 kb	ABAYE2324-ABAYE2325, 2.17 kb	-
PEN	-	ABAYE1696-ABAYE1713, 20.60 kb	-
PSG	-	ABAYE1722-ABAYE1786, 63.24 kb	-
PAA	-	ABAYE2363-ABAYE2376, 13.74 kb	-
PHN	-	ABAYE2317-ABAYE2323, 8.09 kb	-
PCA	ACIAD1702-ACIAD1712, 9.06 kb	ABAYE1673-ABAYE1690, 18.01 kb	ABSDF2016-ABSDF2024, 7.92 kb
QUI	ACIAD1713-ACIAD1716, 6.30 kb	ABAYE1682-ABAYE1685, 6.20 kb	ABSDF2011-ABSDF2014, 5.81 kb, pseudo
SAL	ACIAD1424-ACIAD1427, 4.24 kb	-	-
SOX	ACIAD2549-ACIAD2552, 5.17 kb	-	-
SEU	-	ABAYE2377-ABAYE2390, 16.05 kb	-
ATS	ACIAD1586-ACIAD1601, 20.04 kb	-	-
TAU	-	ABAYE2209-ABAYE2212, 3.60 kb	-
CAI1-R	-	ABAYE2267-ABAYE2295, 30.43 kb	-
CAI2-R	-	ABAYE2418-ABAYE2437, 19.77 kb	-
URE	ACIAD1088-ACIAD1096, 6.34 kb	ABAYE2772-ABAYE2779, 5.76 kb	ABSDF2369-ABSDF2378, 7.20 kb
VAN	ACIAD0978-ACIAD0988, 10.52 kb	ABAYE2621-ABAYE2625, 5.91 kb	-
ComA	ACIAD2639-ACIAD2639, 2.38 kb	ABAYE0884-ABAYE0884, 2.43 kb	ABSDF0886-ABSDF0887, 1.89 kb, pseudo
ComBCEF	ACIAD3314-ACIAD3318, 7.01 kb	ABAYE0316-ABAYE0320, 6.60 kb	ABSDF0325-ABSDF0325, 0.43 kb, pseudo
ComMNOLQ	ACIAD3355-ACIAD3360, 5.11 kb	ABAYE0290-ABAYE0294, 5.17 kb	ABSDF0293-ABSDF0300, 5.17 kb, pseudo
ComP	ACIAD3338-ACIAD3338, 0.44 kb	-	-
PilDCB	ACIAD0360-ACIAD0362, 3.85 kb	ABAYE3444-ABAYE3446, 3.83 kb	ABSDF3191-ABSDF3194, 3.23 kb, pseudo
PilUT	ACIAD0911-ACIAD0912, 2.22 kb	ABAYE2918-ABAYE2919, 2.18 kb	ABSDF2534-ABSDF2537, 2.26 kb, pseudo
AbaG1	-	-	ABSDF3319-ABSDF3341, 20.63 kb
AbaR1	-	ABAYE3551-ABAYE3668, 87.74 kb	-
HEM	ACIAD1458-ACIAD1479, 20.80 kb	-	ABSDF2280-ABSDF2288, 9.37 kb
FUR	ACIAD0910-ACIAD0910, 0.44 kb	ABAYE2920-ABAYE2920, 0.44 kb	ABSDF2538-ABSDF2538, 0.44 kb
TAF	-	ABAYE1887-ABAYE1889, 2.33 kb	-
SID-A	ACIAD2760-ACIAD2779, 27.48 kb	-	-
SID1-R	-	ABAYE1085-ABAYE1104, 26.48 kb	-
IUT	ACIAD0507-ACIAD0509, 1.83 kb	ABAYE3318-ABAYE3320, 1.82 kb	ABSDF3056-ABSDF3058, 1.82 kb
IUT-RS	-	ABAYE2047-ABAYE2051, 2.53 kb	ABSDF1818-ABSDF1822, 5.31 kb
IUT-S	-	-	ABSDF2298-ABSDF2300, 1.82 kb
IUT1-A	ACIAD1528-ACIAD1534, 9.12 kb	-	-
IUT2-A	ACIAD1588-ACIAD1597, 12.40 kb	-	-
RPH1-A	ACIAD1835-ACIAD1865, 18.83 kb	-	-
RPH1-R	-	ABAYE0532-ABAYE0574, 31.33 kb	-
RPH1-S	-	-	ABSDF0699-ABSDF0710, 9.03 kb
RPH2-A	ACIAD2108-ACIAD2203, 75.32 kb	-	-
RPH2-R	-	ABAYE1225-ABAYE1274, 34.76 kb	-
RPH2-S	-	-	ABSDF0997-ABSDF1059, 42.52 kb
RPH3-R	-	ABAYE1830-ABAYE1855, 17.84 kb	-
RPH3-S	-	-	ABSDF1320-ABSDF1337, 8.58 kb
RPH4-R	-	ABAYE2495-ABAYE2548, 37.05 kb	-
RPH4-S	-	-	ABSDF1747-ABSDF1815, 44.59 kb
RPH5-R	-	ABAYE2683-ABAYE2757, 52.46 kb	-
RPH5-S	-	-	ABSDF2443-ABSDF2517, 49.62 kb
RPH6-R	-	ABAYE2875-ABAYE2904, 27.14 kb	-
RPH6-S	-	-	ABSDF2585-ABSDF2616, 20.75 kb
RPH7-S	-	-	ABSDF2750-ABSDF2803, 40.22 kb
RPH8-S	-	-	ABSDF3534-ABSDF3543, 13.64 kb
QS	-	ABAYE3750-ABAYE3761, 18.7 kb	ABSDF0130-ABSDF0132, 1.4 kb, pseudo

### Cell-cell signaling and related processes: biofilm formation and quorum sensing

In a hospital environment, *Acinetobacter* strains may survive several days under severely dry conditions [18]. Vidal and colleagues [19] reported the ability of the nosocomial pathogen *A. baumannii* biotype 9 ACAB715 to form a biofilm on a glass surface, suggesting that this phenomenon plays a key role for the survival of this bacterium under unfavorable environmental conditions. Since biofilm formation may be associated with resistance to immune host response, many clinically significant bacterial species have been studied for their capacity to form biofilm [20]. The different appendages that have been proved to be involved in biofilm formation are type IV pili, flagella, curli and fimbriae. In *A. baumannii* strain AYE, four putative chaperone usher secretion (*csu*) systems have been identified (Table 2). The first system (ABAYE1319-24) has an organization similar to the locus described by Tomaras and colleagues [21] in *A. baumannii* strain ATCC19606 including the Fur box also detected upstream the gene cluster. Furthermore, three putative surface adhesion protein regions have been detected only in strain AYE (Table 2). Apart from the two *csu* systems previously identified in *A. baylyi* [22], the present comparative genomic analysis led to the conclusion that the cluster ACIAD3332-37 might be a third system. Diverse regulation networks such as Crc (Catabolite repression pathway) [23] or two-component systems [24] regulate biofilm formation. Further experiments are therefore required to unravel the respective roles of ABAYE0143 (Crc), ABAYE0258-0259 (EnvZ/OmpR) and the regions ABAYE0667-0671/ABAYE3534-3535 (type IV pili biogenesis regulators/twitching motility) in biofilm production by *A. baumannii* strain AYE.

In many Gram-negative bacteria, quorum sensing (QS) relies on small diffusible molecules, the N-acylhomoserine lactones (AHL). In response to population density, AHL-mediated QS systems modulate expression of genes involved in various biological functions including biofilm formation [25], antibiotics production [26] or Ti plasmid conjugal transfer [27]. AHLs are also known to alter gene expression in host cells [28]. A genomic region (ABAYE3750-ABAYE3761) of strain AYE contains an AHL synthase (ABAYE3761) neighboring a LuxR family transcriptional regulator autoinducer (ABAYE3758). Interestingly, this region has been disrupted by an IS*Aba7* copy in strain SDF and completely lacks in *A. baylyi*. Although strain AYE certainly uses QS modulation, further experiments are required to determine in which broader biological process this system takes part. Nevertheless, additional genes found in the QS genomic region provide some clues. They correspond to genes encoding a Non Ribosomal Peptide Synthase (NRPS) coupled with an efflux pump. NRPS systems are known to produce a large family of natural products like toxins, siderophores, antibiotics or pigments.

Degradation of AHLs may be of medical relevance since AHLs-mediated QS systems *i)* are widespread and highly conserved in many pathogenic bacteria and *ii)* control the expression of genes involved in pathogenicity *sensu lato*. For instance, Whitehead and colleagues [29] hypothesized that the disruption of AHLs-mediated QS systems could possibly help to control diseases. Although the ultimate goal is not clear (competition with other species, neutralization of antibiotics produced by other species…), some bacterial species are able to disrupt a QS signal by the mean of a natural AHL lactonase (see for example [30]). Since other *Acinetobacter s*trains produce AHLase (see for instance [31]), genes coding for similar proteins were searched for. This analysis was difficult since *i)* the similarity between known AHLases is low (ca. 25% identity); *ii)* a part of the motif that characterizes AHLase (HXHXDH) is shared by several unrelated families of proteins (metallo-b-lactamase, glyoxalase II and arylsulfatase). Nevertheless, we were able to find a candidate in those three species (ABAYE0825, ABSDF0820 and ACIAD0766; Figure S2).

### Metabolism: different gene content for different lifestyles

Extensive metabolic pathway reconstruction was undertaken for the three *Acinetobacter* strains. As reported in *A. baylyi* str. ADP1 [9], the central metabolism study of both *A. baumannii* strains confirms the lack of several key enzymes: a glucokinase, proteins of a phosphotransferase transport system, a 6-phosphofructokinase and a pyruvate kinase. The latter activity which exists in *Pseudomonas* spp. seems to be a good marker to clearly delineate both genera. Few *Acinetobacter* isolates are able to grow on glucose as sole carbon source and this property was early used as a main criterion to classify *Acinetobacter* species [32]. The Embden–Meyerhof–Parnas glycolysis can certainly not be performed in *Acinetobacter* spp. given the lack of the previously mentioned enzymes. An alternative glycolytic route passing through the Entner-Doudoroff pathway was elucidated several decades ago [33]. It starts with glucose periplasmic oxidation and leads to pyruvate formation. All the genes required for this pathway were identified in the three Acinetobacter genomes. Interestingly, *A. baylyi* and *A. baumannii* strain SDF only possess the *gcd* gene which encodes a membrane-bound quinoprotein glucose dehydrogenase [34] whereas strain AYE has two additional genes (ABAYE1605, ABAYE1751) which encode soluble forms of quinoprotein dehydrogenases (s-GDH) [35] thus possibly allowing the utilization of alternative substrates by the cells.

The three studied strains are prototrophic and are able to grow using succinate as sole carbon source. Among the 499 genes of *A. baylyi* which are essential for life on minimal medium [36], more than 90 percent have homolog in both *A. baumannii* genomes. Consitent with this high level of conversation, the predicted amino acid biosynthetic pathways of the AYE and SDF strains are similar to those previously described in *A. baylyi* [9]. In strain SDF, almost all genes involved in the biosynthesis of main cell components were kept intact except the *trpE* pseudogene (ABSDF3251) which normally encodes the first subunit of the anthranilate synthase, an essential enzyme for tryptophan biosynthesis. However, this deficiency may be compensated by two genes (ABSDF3335, ABSDF3336) which encode the two anthranilate synthase subunits and are located in the AbaG1 island [10]. This genomic region is likely to be under strong selective pressure to maintain a tryptophan prototrophy. Despite its genome reduction (pseudogenization and gene loss), strain SDF successfully maintained a prototrophic lifestyle.

In contrast to *A. baylyi*, both *A. baumannii* strains genomes harbor the *cobUTCS* genes (Table 2). This is a major difference in terms of biosynthesis capabilities since these genes encode the necessary enzymes for the last steps of adenosyl-cobalamin (coenzyme B12) synthesis starting from dimethylbenzimidazole and cobinamide as precursors [37]. However, this pathway is probably not functional anymore in strain SDF because *cobS* and *cobT* genes are truncated. Only two reactions requiring coenzyme B12 as cofactor have been detected in the *A. baumannii* str. AYE genome. The first is a methionine synthase encoded by the gene *metH* (ABAYE2822) which also exists in *A. baylyi*. This B12-dependent reaction allows *Acinetobacter* to synthesize methionine via a non-essential alternative route. The second is the ethanolamine ammonia-lyase which is essential for ethanolamine catabolism. The two subunits of the corresponding protein are encoded by the *eutB* (ABAYE1458) and *eutC* (ABAYE1457) genes in strain AYE. These genes are missing in *A. baylyi* and *eutC* is disrupted in strain SDF. The catabolism of ethanolamine by strain AYE may be an important carbon and nitrogen sources during the infection process since ethanolamine is a major constituent of cell membrane phospholipids. Furthermore, this compound is a common excipient used in drug solutions.

*A. baylyi* is a nutritionally versatile soil bacterium. Its genome analysis revealed that a large amount of genes (10%) are dedicated to the catabolism of a large variety of compounds [9], 38. Most of these genes are clustered in several genomic regions called catabolic islands which are themselves located side by side in an archipelago accounting for one-quarter of the overall genome (Figure 3). The comparison between strain AYE and *A. baylyi* revealed a notable conservation of catabolic capacity (Table 2). Furthermore, strain AYE islands are also clustered on the chromosome (i.e. around the terminus of replication and within the third quarter of the genome, Figure 3). The *dca-pca-qui-pob-hca* chromosomal cluster of *A. baylyi* encodes enzymes that degrade dicarboxylic acids and aromatic compounds [39]. Interestingly, many of these compounds are components of suberin, a protective polymer which is produced by plants in response to stress. In strain AYE, all these genes are also present supporting the hypothesis that soil is also the primary niche of *A. baumannii* species. Nevertheless, the local organization of these islands is different between both strains. Only the *pca-qui* genes have been maintained together in strain AYE. This observation is in agreement with experimental results performed in *A. baylyi* reporting a possible *pca-qui* operon structure of 14-kb [40]. As an alternative in strain AYE, *hca* and *van* genes are gathered and may improve ferulic acid degradation leading to vanillate production which is subsequently metabolized by the *van* genes [41]. Different selective pressures may have shaped the genomic island organization in accordance to available nutrients in their natural environment. In addition, *A. baumannii* str. AYE harbors nine new gene clusters that are absent in *A. baylyi* (Table 2). Three of them, called CAI1-R, SEU and CAI2-R, form large-size genomic islands of 30.4-kb, 16-kb and 19.8-kb respectively. These regions contain a high proportion of genes encoding enzymes like oxygenases and need to be deeply investigated in order to determine their exact catabolic role. Other AYE specific regions have been assigned a more precise function.

The CBE cluster of strain AYE may encode genes for choline degradation to produce betaine. In contrast to *A. baylyi*, *sox* genes are missing in strain AYE and are necessary for betaine catabolism. Presumably, betaine is not a carbon source for strain AYE and is accumulated in the cytoplasm. In response to osmotic stress, high concentrations of glycine betaine would act as an osmoprotectant [42]. In addition, this molecule is also a potent protector against mutagenic compounds and radiation-induced damages. As ethanolamine, choline is a main component of cell membrane phospholipids.

The TAU cluster of strain AYE contains genes encoding a taurine transporter and the dioxygenase TauD. Like *Escherichia coli* K12 [43], strain AYE could utilize taurine as a sulfur source when facing sulfate starvation conditions. Taurine plays several important roles in mammals: neuromodulation, bile acid conjugation, detoxification, osmoregulation, membrane stabilization, and regulation of intracellular Ca^2+^ homeostasis [44]. Competition for taurine use might take place during *A. baumannii* infection process.

Finally, the PEN genomic region of strain AYE chromosome contains fifteen genes which are likely to be involved in penicillin catabolism providing carbon and nitrogen to the bacteria. One of them (ABAYE1713) encodes a putative penicillin acylase (Pac). The substrate specificity of this acylase is still unknown in strain AYE. As reported in *E. coli* str. W [45], the *pac* gene is positively regulated by the PaaX repressor of phenylacetate catabolism. Pac could be a scavenger enzyme for natural compounds containing a phenylacetate residue or derivative [46].

### Iron uptake systems: toward a high degree of specialization

One possible defense mechanism of human host against bacterial infection is the reduction of free extracellular iron concentration via iron-binding proteins (lactoferrin, transferrin). Indeed, normal concentration of free iron in the body oscillates around 10^−18^ M, whereas the minimal concentration required for the bacterial survival is ca. 10^−6^ M [47]. Iron/heme acquisition can be achieved thanks to direct contact between the bacterium and the exogenous iron/heme sources. Bacteria may also take out iron from their environment using high affinity iron molecules called siderophores/hemophores that are released outside cells.

*A. baumannii* strain AYE may chelate iron using a siderophore (ABAYE1085-ABAYE1104) that can compete with host iron-binding proteins to overcome the iron starvation imposed by the host. The same siderophore (acinetobactin) has been described in another *Acinetobacter* strain (ATCC19606; [48]), which was structurally related to the anguibactin of the fish pathogen *Vibrio anguillarum* [49]. One of the key compounds of these siderophores is the histamine which results from histidine decarboxylation. Tolmasky and colleagues [50] showed that when *V. anguillarum* cells are cultured on 1% histidine, a histidine decarboxylase activity is detected, leading to the production of histamine. Likewise, *A. baumannii* strain ATCC19606 produces histamine when grown with histidine [51]. As expected, we found a histidine decarboxylase gene (*hdc*) located within the iron uptake region in strain AYE (ABAYE1098). Interestingly, *A. baylyi* genome has a siderophore gene which composition is not based on histamine because this species lacks the *hdc* gene [9].

The transport of the ferri-siderophore (iron-siderophore complex) into the cell is performed by the Iron Regulated Outer Membrane Proteins system (IROMPs). This IROMPs system is typically made of one specific outer-membrane receptor, one periplasmic protein and several inner-membrane-associated proteins acting either as receptors or as energy transducers (*i.e.* the ExbB:ExbD:TonB complex). The strain AYE potentially possesses two similar uptake systems (ABAYE2046-2051 and ABAYE3318-3320). Once internalised, the ferri-siderophore is reduced to release iron by an enzyme having a ferric reductase activity. We did not identify any structurally related protein. However, Fontecave et al. [52], have reported that several enzymes (nitrite reductase, sulfite reductase…) which are not necessarily tightly linked to iron metabolism could present a ferric reductase activity. Thus, a possible candidate is the gene tandem ABAYE1544-ABAYE1545 which encodes the two subunits (large and small) of the nitrite reductase.

Surprisingly, siderophores were not identified in strain SDF. Since iron is necessarily required for life, strain SDF must use a different strategy to scavenge iron from its environment. Several evidences suggest that A. *baumannii* strain SDF is able to use haem or haemoglobin as source of iron. *A*. *baumannii* SDF is in close contact with the gut of haematophage organisms [53] and therefore is readily in contact with blood cells. In addition, we found in the genome of this strain several haemagglutinin/haemolysin-related genes encoding proteins that can aggregate erythrocytes and destroy their membrane releasing haemoglobin. We located also a gene cluster (ABSDF2280-88) sharing similarities with genes associated with haem acquisition system (HAS) already described in several species (reviewed in [54]). According to the literature the transport system of haem or haemoglobin into the cell could also be ExbB:ExbD:TonB complex dependant but in this case the periplasmic intermediary is not required [55]. Several clusters (N = 3) of ExbB, ExbD, and TonB genes have been detected on strain SDF genome as well as several associated ABC transporters which are probably involved in the translocation of haem/haemoglobin from the periplasm to the cytosol. Finally, the key enzyme (Haem Oxygenase HemO = ABSDF2281) required for the release of iron has also been found in this strain.

Bacteria generally regulate iron import into the cell according to iron availability. In both AYE and SDF strains, the regulation of this process is likely to be mediated by the ferric regulator uptake protein (Fur: ABAYE2920 and ABSDF2538) which acts as a transcription repressor of genes involved either in the siderophore synthesis, in transport or even in degradation of ferri-siderophore/haemo(globin). Furthermore, we identified *fur* boxes in the upstream region of most of the gene clusters involved in iron capture. Besides the central role of Fur protein, secondary positive regulators may also interfere in the iron uptake process. In *Vibrio anguillarum*, the assembly of the siderophore anguibactin and the enhancement of iron transport are promoted by products encoded in a transacting factor region of the virulence plasmid pJM1 [56], [57].

The present comparison of iron uptake provides a good example of specialization of *A. baumannii* strains facing different environmental conditions. In the case of *A. baumannii* strain AYE, the competition against human host for iron using siderophores may explain part of the pathogenicity of this strain. In contrast, strain SDF may not have to compete for iron and hence use an alternate system.

### Acinetobacter pathogenicity: insights into virulence factors

Gene products which give a microbe the ability to persist and invade a host of particular species are often referred as virulence factors. These factors generally comprise toxins, cell surface components (adhesins, glycoproteins), and hydrolytic enzymes that may contribute to the pathogenicity of the bacterium. Since no toxin or toxin-like encoding gene has been detected in both *A. baumannii* strains, their pathogenicity relies essentially on genes which belong to the two last categories.

Outer membrane proteins (OMPs) of Gram-negative bacteria are known to have essential roles in antibiotic resistance as well as in adaptation and pathogenesis in host cells [58]. Several OMPs of the OmpA family were characterized in various *Acinetobacter* strains and represent one of the major OMP in this genus. The *A. baylyi* OmpA is a secreted protein having an emulsifying activity [59] whereas other OmpA proteins are associated to virulence properties which induce apoptosis of epithelial cells [60] or stimulate gastrin and interleukin-8 gene expression [61]. The present genome analysis reveals that only one gene in each *Acinetobacter* strains encodes an OmpA protein (ACIAD0697, ABAYE0640, and ABSDF0605). These proteins share more than 89% of identical amino acids and have the same chromosomal context. These findings suggest that OmpA proteins are ubiquitous in the *Acinetobacter* genus but also raise questions on their exact role.

Among hydrolytic enzymes, phopholipases have been the subject of extensive investigation, especially the two types of phospholipases C produced by *Pseudomonas aeruginosa*: PLC-H encoded by the *plcS* gene is acidic and has a strong hemolytic activity whereas its counterpart PLC-N encoded by the *plcN* gene is rather basic and does not have any hemolytic activity (for a review see [62]). PLC-H is actually posttranslationnally altered by one of the *plcR* gene product thus enhancing its hydrolytic capacity [62]. We found in each *Acinetobacter baumannii* genome two copies of phospholipase C (*plc*) genes sharing about 50 percent identity (Table S1). This suggests that the proteins arose from an ancient duplication. Interestingly, though each copy has its best BlastP match with PLC-N, each protein has a pI lower than 7 which is rather a feature of PLC-H. We therefore cannot conclude on the hemolytic activity of *A. baumannii* phopholipases C. Furthermore, no *plcR* gene homolog has been found in both *A. baumannii* strains. Whether *plcR* is required for PLC-H activity is not clear, the hemolytic property of *plc* genes products will therefore remain an open question.

As other phospholipases (D, A) are thought to be major virulence factors in other species (see for example [63]), we have checked for their presence in the genomes under study. We effectively found one or several copies of the two kinds of phospholipases. The most interesting case is certainly the *pld* gene copies harbored by the AYE genome. Among the 4 copies, 3 displayed peculiar features. They *i*) were bordered at both sides by an insertion sequence (IS*Aba1*); *ii*) were identical indicating that the amplification of the *pld* copies is rather recent; *iii*) presented a slippery sequence (GGGGG AAC CUU) resulting in a +1 frameshift analogous to the one found in the *prfB* gene of *E. coli*. Given these evidences, we speculate that the intact copy of *pld* maintains a basal activity that is necessary for the cell to exploit phosphorous sources whereas the 3 remaining ones are likely to be active but solicited only when facing exceptional environmental conditions (infection process, etc.). Indeed, the strong promoter provided by IS*Aba1* and the programmed frameshift would allow the regulation of the production of fully functional phospholipase D.

While this work was in progress, the sequence of another *A. baumannii* (strain ATCC17978) was reported [64]. In this human-adapted *A. baumannii* isolate, the virulence factors previously described in strain SDF and AYE were also present. Moreover, in this study, candidate virulence genes were identified by insertional mutagenesis followed by two screening assays (reduced brood size of *C. elegans* and inhibition of *D. discoideum*) in which *A. baumannii* ATCC17978 virulence was primarily induced by 1% ethanol [65]. Among the 35 Ethanol-stimulated virulence (Esv) mutants retained by Smith and colleagues, 14 (16 Esv genes are actually reported in the Genbank file) apparently did not have any homolog in *A. baylyi*. However, after confronting their data and ours, we noticed that actually only 5 Esv genes did not have any correspondence in *A. baylyi* including 2 from phage origin and 3 putative transcriptional regulators (Table 3). Still according to our annotation, many of the candidate virulence genes were either involved in main cell functions (DNA repair, general metabolic functions, etc.) or proved to be essential in other species thus raising doubts on their effective inactivation by the transposon insertion.

**Table 3:** Functional annotation proposed for 16 Esv-mutants published by smith and colleagues [64].

Gene label^a^	Gene Name	Esv-mutant Name^a^	Annotation	Putative Ortholog	Essential in *A. baylyi* ADP1^b^	Essential in *E. coli* c
A1S_3223	-	EsvA	putative transcriptional regulator		No Homolog	No Homolog
A1S_1232	-	EsvB	putative transcriptional regulator	ABAYE2526	No Homolog	No Homolog
A1S_1012	*ureA*	EsvC	urease gamma subunit	ABAYE2778,ABSDF2377,ACIAD1089	No	No
A1S_2447	*pstC*	EsvD	high-affinity phosphate transport protein	ABAYE1033,ABSDF1101,ACIAD1213	No	No
A1S_2313	*queF*	EsvE1	7-cyano-7-deazaguanine reductase	ABAYE1164,ABSDF1213,ACIAD2261	No	No
A1S_2314	-	EsvE2	conserved hypothetical protein	ABAYE1163,ABSDF1212,ACIAD2262	No	No
A1S_2315	*rodA*	EsvE3	rod shape-determining protein	ABAYE1162,ABSDF1211,ACIAD2263	No	Yes
A1S_3218	*czcB*	EsvF1	RND divalent metal cation efflux membrane fusion protein	ABAYE0270,ABSDF0264,ACIAD3376	No	No
A1S_3290	-	EsvF2	putative transcriptional regulator	ABAYE0200,ABSDF3398,ACIAD3452	No	No
A1S_0378	*etfD*	EsvG	electron transfer flavoprotein-ubiquinone oxidoreductase	ABAYE3397,ABSDF3133,ACIAD3259	No	No
A1S_2262	*rpoH*	EsvH	sigma H (sigma 32) factor of RNA polymerase	ABAYE1217,ABSDF1264,ACIAD1311	Yes	Yes
A1S_2037	-	EsvI	putative transcriptional regulator		No Homolog	No Homolog
A1S_3329	-	EsvJ	conserved hypothetical protein	ABAYE0156,ABSDF3464,ACIAD3508	No	No
A1S_1586	-	EsvK1	putative phage endonuclease		No Homolog	No Homolog
A1S_1587	-	EsvK2	putative phage Terminase		No Homolog	No Homolog
A1S_0310	*uvrC*	EsvL	excinuclease ABC subunit C	ABAYE3465,ABSDF3216,ACIAD0340	No	No

Automatic functional annotation was first generated using our annotation pipeline [71] subsequently reviewed by human expert.

a taken from [64],

b taken from [36],

c data from *E. coli* chromosome (PEC) database (http://www.shigen.nig.ac.jp/ecoli/pec/index.jsp).

Although additional investigations are needed to demonstrate the presence of true virulence genes in *A. baumannii*, the present work provides keys for understanding the adaptability of Acinetobacters to various ecological niches.

## Materials and Methods

### Sequencing

The complete genome sequences of *A. baumannii* strains AYE and SDF were determined using the whole-genome shotgun method. Two libraries, obtained by chemical shearing of genomic DNA and cloning of generated 2–5 kb and 6–17 kb inserts into the plasmids pNAV (A) and pCNS (B) (pcDNA2.1 and pSU18 derived, respectively), were constructed per genome. DNA Plasmids were purified and end-sequenced (61440 (A) and 20928 (B) for AYE and 62784 (A) and 22272 (B) for SDF) by dye-terminator chemistry on ABI3730 sequencers (Applied Biosystems, Foster City, United States). All reads were first compared to *A. baylyi* ADP1 rRNA (16S and 23S sequences) and ISfinder database [66], and those sharing at least 80% identity on a length of 100 nt were tagged as rRNA sequences (C) or ISs (D). A first assembly for each genome, using Phred/Phrap/Consed software package (www.phrap.com), was performed without 713 (C) and 2691 (D) reads for AYE and 514 (C) and 18156 (D) reads for SDF. Repeated sequences longer than 600 nt were detected with the RepSeek software [67] and subsequently recorded to an internal database. Similarity searches across the newly created database using same criteria as mentioned above, allowed the identification of 1699 and 2434 reads as part of repeated sequences in AYE and SDF, respectively. A new assembly was constructed without rRNA, IS and internal repeated sequences and rRNA. IS and internal repeated reads were then reinserted in the assembly on the clone-links basis. Additional reactions were necessary to complete the genomic sequences of *A. baumannii* strains AYE and SDF (9648 and 19240, respectively).

### Annotation and Comparative Genome Analyses

CoDing Sequences were first predicted using the AMIGene (Annotation of Microbial Genomes) software [68]. Each CDS was then submitted to automatic functional annotation. Putative orthologous relations between two genomes are defined as gene pairs that satisfy either the Bidirectional Best Hit criterion [69] or an alignment threshold (at least 40% sequence identity on at least 80% of the length of the smallest protein). These relations are subsequently used to search for conserved gene clusters (synteny group) among several bacterial genomes. The algorithm based on an exact graph-theoretical approach is detailed in [70]. This method allows for *i)* multiple correspondences between genes and, thus, paralogy relations and/or gene fusions are easily detected; *ii)* Chromosomal rearrangements (inversion, insertion/deletion). A ‘gap’ parameter, representing the maximum number of consecutive genes which are not involved in a synteny group, was set to five.

As a final step, manual validation of automatic annotations was performed using the MaGe web interface [71].

### Data Availability

Annotation sets of all *A. baumannii* replicons have been deposited in EMBL database under the following references:

*Acinetobacter baumannii* str. SDF: Chromosome, CU468230; Plasmid 1, CU468231; Plasmid 2, CU468232; Plasmid 3, CU468233.

*Acinetobacter baumannii* str. AYE: Chromosome, CU459141; Plasmid 1, CU459137; Plasmid 2, CU459138; Plasmid 3, CU459140; Plasmid 4, CU459139.

These annotations as well as comparisons results are publicly available for consultation at http://www.genoscope.cns.fr/agc/mage.

## Supporting Information

Figure S1 Phylogenetic tree reconstruction for all ISRs from *Acinetobacter spp.* available in Genbank (release 160), using as outgroup *Escherichia coli* BCRC 15486 ISR. This analysis shows that *A. baumannii* strain SDF is effectively a baumannii species although it is devoid of *ampC* gene and is unable to grow at 44°C (two phenotypic traits that characterize species belonging to the *A. calcoaceticus*-*A. baumannii* complex). Moreover, it confirms the closeness of the relationship between *A. calcoaceticus*, *A. baumannii* and genomic species 3 and 13TU. Sequences were first aligned with MUSCLE [72] and the PhyML software [73] was used to build the tree (substitution model: HKY, 100 bootstrap replicates).
(1.52 MB TIF)

Figure S2 Alignment of presumed AHL lactonases of the 3 Acinetobacters under study together with other known AHL lactonases from *Bacillus subtilis*, *Bacillus thurengiensis*, *Arthrobacter sp.* and *Agrobacterium tumefaciens*. The red boxes indicate active residues of AHLases. Alignment was performed with MUSCLE [72] embedded in JalView [74].
(2.43 MB TIF)

Table S1 Labels of genes annotated as (putative) phospholipase in four *Acinetobacter* genomes. Frameshifted copies are indicated in bold type (part1–part2).
^a^Data from [64].
(0.02 MB XLS)
