# Supplementary figures and images for: Comparative Analysis of Acinetobacters: Three Genomes for Three Lifestyles
Source: PLoS One. 2008 Mar 19;3(3):e1805. doi: 10.1371/journal.pone.0001805 (PMC2265553; doi:10.1371/journal.pone.0001805)

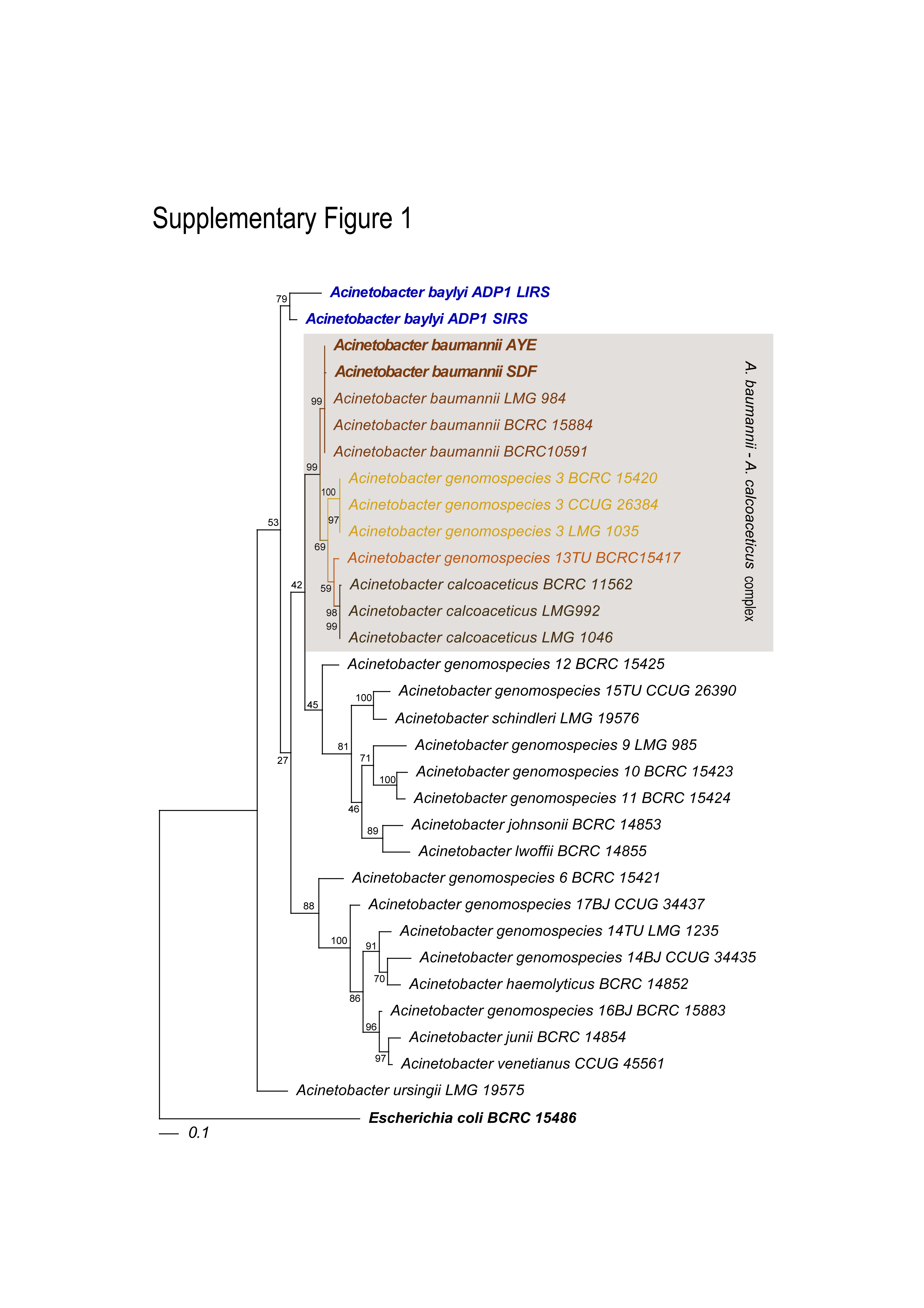

Supplement: Figure S1 — Phylogenetic tree reconstruction for all ISRs from Acinetobacter spp. available in Genbank (release 160), using as outgroup Escherichia coli BCRC 15486 ISR. This analysis shows that A. baumannii strain SDF is effectively a baumannii species although it is devoid of ampC gene and is unable to grow at 44°C (two phenotypic traits that characterize species belonging to the A. calcoaceticus-A. baumannii complex). Moreover, it confirms the closeness of the relationship between A. calcoaceticus, A. baumannii and genomic species 3 and 13TU. Sequences were first aligned with MUSCLE [72] and the PhyML software [73] was used to build the tree (substitution model: HKY, 100 bootstrap replicates). (1.52 MB TIF) [file pone.0001805.s001.tif]

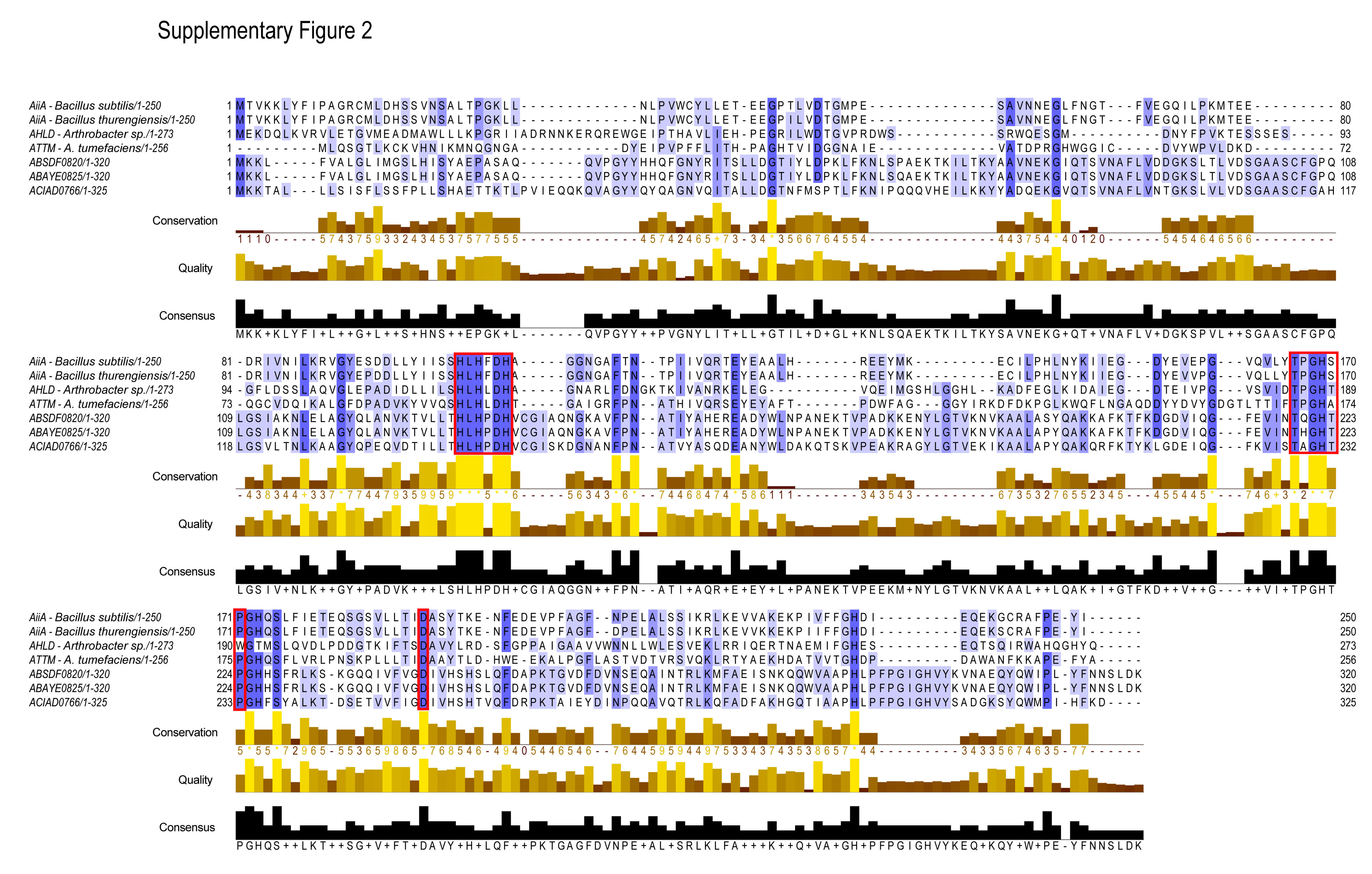

Supplement: Figure S2 — Alignment of presumed AHL lactonases of the 3 Acinetobacters under study together with other known AHL lactonases from Bacillus subtilis, Bacillus thurengiensis, Arthrobacter sp. and Agrobacterium tumefaciens. The red boxes indicate active residues of AHLases. Alignment was performed with MUSCLE [72] embedded in JalView [74]. (2.43 MB TIF) [file pone.0001805.s002.tif]
